# St. Vincent’s Hospital, New York City

**Published:** 1849-12

**Authors:** 


*St. Vincent's Hospital, New York City.*—A short time since, the attention of our readers was invited to a circular announcing the institution of a new hospital at Philadelphia—St. Joseph's Hospital—organized upon the plan of the Buffalo Hospital of the Sisters of Charity. Within a few days we have noticed, in one of the New York papers, the card of a new hospital in that city, which was opened on the 12th ult., under the charge of the Sisters of Charity. The latter hospital is on the plan of that in Buffalo, the regulations, indeed, being not only similar in substance, but even in language.

The Medical and Surgical officers of the St. Vincent's Hospital, (which is the name given to that just referred to,) are as follows:

*Attending Physicians*—Wm. Power, M. D., Wm. Murray, M. D.

*Visiting Physician*—Richard Fraser, M. D.

*Attending Surgeons*—Wm. H. VanBuren, M. D., John W. Schmidt, Jr., M. D.

*Consulting Surgeon*—Valentine Mott, M. D.

We have reason to believe that the successful operation of the Buffalo hospital furnished the suggestion for the establishment of the new hospitals at Philadelphia and New York City.

